# Peripheral blood BDNF and soluble CAM proteins as possible markers of prolonged disorders of consciousness: a pilot study

**DOI:** 10.1038/s41598-023-50581-8

**Published:** 2024-01-03

**Authors:** L. Coppola, G. Smaldone, A. M. Grimaldi, A. Estraneo, A. Magliacano, A. Soddu, G. Ciccarelli, M. Salvatore, C. Cavaliere

**Affiliations:** 1 IRCCS Synlab SDN, Naples, Italy; 2 Istituto Di Ricovero e Cura a Carattere Scientifico (IRCCS) Fondazione Don Carlo Gnocchi, Florence, Italy; 3 Department of Physics and Astronomy, Western Institute of Neuroscience, University of Western Ontario, London, ON Canada

**Keywords:** Neuroscience, Neurology, Neurological disorders

## Abstract

Although clinical examination still represents the gold standard for the differential diagnosis of prolonged disorders of consciousness (pDoC), the introduction of innovative markers is essential for diagnosis and prognosis, due to the problem of covert cognition. We evaluated the brain-derived neurotrophic factor protein (BDNF) and the soluble cell adhesion molecules proteins (CAMs) in a cohort of prolonged disorders of consciousness patients to identify a possible application in the clinical context. Furthermore, peripheral blood determinations were correlated with imaging parameters such as white matter hyperintensities (WMH), cranial standardized uptake value (cSUV), electroencephalography (EEG) data and clinical setting. Our results, although preliminary, identify BDNF as a possible blood marker for the diagnosis of pDoC (p value 0.001), the soluble CAMs proteins CD44, Vcam-1, E-selectin (p value < 0.01) and Icam-3 (p value < 0.05) showed a higher peripheral blood value in pDoC compared with control. Finally, soluble Ncam protein could find useful applications in the clinical evolution of the pDoC, showing high levels in the MCS and EMCS subgroups (p value < 0. 001) compared to VS/UWS.

## Introduction

Comatose patients suffering from acquired severe brain injury can evolve in vegetative state/unresponsive wakefulness syndrome (VS/UWS) in which they are awake but unconscious; some of them recover minimal but reproducible behavioral signs of consciousness and are classified in minimally conscious state (MCS), or recover full consciousness and can be classified as emerged from a minimally conscious state (EMCS)^1,2^. A correct diagnosis is critical for predicting clinical outcomes, as patients in MCS are more likely to recover full consciousness than patients in VS/UWS. To improve diagnostic accuracy, clinicians have tools recommended in recent internationally shared by American Academy of Neurology and European Academy of Neurology guidelines, such as the Coma Recovery Scale-Revised (CRS-R)^3^. However, it is known that clinical assessment alone is not sufficient to detect cases of covert cognition^4^. Therefore, it is necessary to evaluate and introduce innovative assessment criteria that could better characterize both the diagnosis and prognosis of patients with pDoC. Recently, blood levels of neurodegeneration markers such as neurofilament light chain (NF-L)^5^, glial fibrillary acidic protein (GFAP)^6^ have been found higher in patients with pDoC compared to the Healthy Controls (HCs), and correlated with neuroimaging data^7^. Based on these preliminary findings, additional blood markers of neuronal impairment (i.e. BDNF and soluble cell adhesion molecules, CAMs) could be assessed to better profile pDoC patients.

The BDNF protein is a protein primarily expressed in the brain and central nervous system; it plays a primary role in the growth, development, and maintenance of nerve cells, as well as in the regulation of synaptic plasticity^8^; its expression changes after acute brain injury, suggesting a potential role in a neuronal and synaptic reorganization after brain injury. The peripheral blood BDNF levels after acute brain injury in humans have been poorly evaluated^9^; CAMs proteins are involved in cell adhesion, the process by which cells come together through the interaction of certain proteins. These proteins are released into the bloodstream upon activation or damage^10^. CAMs include members of the selectin family, such as P-selectin, E-selectin, and L-selectin, and the integrin family, such as soluble intercellular adhesion molecule-1 (sIcam-1) and soluble vascular cell adhesion molecule-1 (sVcam-1)^11^. They play crucial roles in different physiological and pathological processes, such as inflammation and immune responses. Therefore, their peripheral blood detection can be used as a biomarker of different diseases or conditions, such as cardiovascular diseases, multiple sclerosis, and acute neurological diseases^12,13^. CAMs proteins play an important role in the traumatic brain injury (TBI)^14^; following TBI, there is an increased expression of CAM proteins, which are known to play key roles in the inflammatory response, neural repair, and regeneration. They also are involved in the regulation of neuroinflammatory processes and the recruitment of immune cells to the site of injury^15^; indeed, their alterations in terms of expression and function may therefore contribute to cognitive impairment following TBI. Because of their involvement in various processes related to neural repair and inflammation, we have deepened their study to understand the processes of neurodegeneration and repair^16^. In this scenario, the present observational study aimed at investigating the possible diagnostic and/or prognostic role of peripheral blood BDNF protein and soluble CAMs proteins. For these purposes we retrospectively analyzed their blood levels in a cohort of patients with pDoC and investigated their association with patients clinical, neuroimaging (i.e., white matter hyperintensities WMH, cranial SUV cSUV) and electroencephalography (EEG) data.

## Material and methods

### Study population

We analyzed for the retrospective study the plasma samples of pDoC patients (n = 19) who underwent PET-MRI. In the same session, immediately before PET-MRI acquisition, blood samples were collected. The pDoC patients were divided by clinical diagnosis (VS/UWS, MCS, EMCS) and subsequent plasma samples and imaging analyses were also included in the evaluation of the markers. In addition, n = 12 healthy controls (HCs) were enrolled to compare the results obtained on the analyzed markers. UWS, MCS or EMCS diagnostic criteria were used to patients’ selection^17^. Inclusion and exclusion criteria are the same as those used previously^7^.

### Standard protocol approvals, registrations, and patient consents

The study was approved by the local Ethics Committee of IRCCS Pascale (Protocol number: 9/21 date: 16/12/21) and performed according to the ethical principles introduced in 1964 by the Declaration of Helsinki^18^. The legal guardians of the patients signed informed consent. We obtained blood samples from IRCCS SYNLAB SDN Biobank in Naples (Italy), a partner of the European network BBMRI-ERIC.

### Blood collection and ultra‑sensitive determination

Plasma samples were centrifuged at 10,000*g* for 10 min at + 4 °C and BDNF concentration was measured using the commercially available Simoa™ BDNF Discovery Kit using the Simoa SR-X instrument (cat. #102039 Quanterix, Lexington, USA). Plasma samples were diluted as suggested by the kit data sheet. The limit of detection for Simoa™ BDNF Discovery Kit was 0.0072 pg/mL and the lower limit of quantification was 0.0686 pg/mL when compensated for 500× sample dilution. The CV of 20% was accepted as adequate for analyses. The researcher who performed the BDNF assays had no information related to the clinical data. Blood samples from HCs stored in the IRCCS SYNLAB SDN Biobank were obtained and processed in the same manner. The LEGENDplex™ Human Adhesion Molecule Panel multiplex assay (cat. #740945, BioLegend, San Diego, USA) was used to measure the plasma level of the following soluble cell adhesion molecules (CAMs): intracellular CAM 1, 2 and 3 (Icam-1, Icam-2, and Icam-3, respectively), vascular CAM (Vcam-1), platelet endothelial CAM 1 (Pecam-1), activated leukocyte CAM 1 (Alcam-1), epithelial CAM (EpCAM), neural CAM (Ncam), endothelial selectin (E-selectin), platelet selectin (P-selectin), leukocyte selectin (L-selectin), P-selectin glycoprotein ligand-1 (Psgl-1), and CD44.

### PET/MRI acquisition protocol and structural data processing

PET/MRI data were obtained at the simultaneous acquisition using a Biograph mMR tomograph (Siemens Healthcare, Erlangen, Germany) designed with a multi-ring LSO detector block embedded in a 3-T magnetic resonance scanner. The axial and transverse nominal resolution of the PET system was 4.4 and 4.1 mm FWHM, respectively, at 1 cm from the isocenter^19^. 5 MBq/Kg of the ^18^F-fluorodeoxyglucose (^18^F-FDG) tracer was used for dynamic brain PET study. Patients were recommended to do a minimum of 6 h fasting before PET imaging. Blood glucose less than 120 mg/dl was used as a reference for proceeding to FDG injection. The PET-MRI and structural data (WMH, SUV) processing protocol details are previously reported^7,20, 21^. An exemplificative image of PET/MRI acquisition is reported in Supplementary Fig. 1.

### EEG acquisition protocol and data processing

Resting state EEG (rsEEG) was recorded using a portable EEG device (Nicolet video-EEG system). Standard procedure of 30 min eye-closed rsEEG recording was performed. The rsEEG were recorded with a sampling frequency of 512 Hz, bandpass between 1 and 70 Hz with notch filter on. Electrode montage included 19 scalp electrodes placed according to the international 10–20 System (O1, O2, P3, P4, Pz, T5, T6, C3, C4, Cz, T3, T4, F3, F4, Fz, F7, F8, Fp1, and Fp2). A frontal ground electrode was used, while cephalic midline electrode was used as an electric reference according to standards. Electrode impedances were kept below 10 Kohm. Forced eyes closing by cotton wool was employed in the condition of patients awake (spontaneous eye opening). For the EEG pre-processing, the rsEEG were imported in EEGLAB. Each rsEEG was divided into epochs of 2 s and analyzed offline^22^. The EEG epochs containing physiological artifacts (blinking, muscular movements, head movements) and non-physiological (bad contact between electrode and scalp, sweating, etc.) artifacts were discarded by visual analysis. Sleep figures (K-complexes, sleep spindles, etc.) if present were also detected and discarded. Following this first visual check, the remaining artifact-free epochs underwent an ICA decomposition analysis for the residual artifact removal^23^.

### The spectra analysis of rsEEG epochs

Power density of scalp rsEEG rhythms was computed through a digital FFT-based analysis (Welch technique, Hanning windowing function, no phase shift) using the artifact-free epochs mentioned above. The EEG frequency bands of interest were individually identified based on the following frequency landmarks, the transition frequency (TF) and individual alpha frequency peak (IAFp; Klimesch 1999^24^). The TF marks the transition frequency between the theta and alpha bands, defined as the minimum of the rsEEG power density between 3 and 8 Hz (between the delta and the alpha power peak). The IAF is instead defined as the maximum power density peak between 6 and 14 Hz (Klimesch et al. 1996^25^, 1998^26^; Klimesch 1999^24^). The TF and IAF were 6 and 10 respectively for each subject involved in the study. Based on these landmarks, we estimated delta, theta, and alpha bands as follows: delta from TF − 4 Hz to TF − 2 Hz, theta from TF − 2 Hz to TF, and alpha from TF to IAF. Moreover, given the increased electromyographic (EMG) noise resulting from involuntary muscle movements, particularly in MCS patients, we limited our data analysis to the alpha, theta, and delta frequency bands^27^. This restriction was applied to mitigate potential confounding factors when comparing activity in these bands between patients and healthy controls, as our primary focus was on these frequency ranges where EMG noise had minimal influence.

### Data analysis

P values were calculated as described in individual figure legends using Graphpad Prism 7 (Graphpad Software) and considered significant below 0.05. No statistical method was used to predetermine sample size, and experiments were not randomized. All assays were performed in duplicate. The results are reported as mean ± standard deviation of duplicate.

## Results

### Patient characteristics

Table 1 reports pDoC patients clinical and demographic characteristics; the cohort characteristics, such as age, sex, diagnosis (at 0–8 months and at 9–18 months) and etiology are reported for patients with pDoC and HC. Furthermore, as shown in Table 1, subsequent measurements during the follow-up were reported in the etiology section. Indeed, 10 out of 19 pDoC patients were also evaluated with additional measurements within 18 months and included in the final analysis (n = 31).

**Table 1:** Patients characteristics.

Retrospective study (n = 31)	
pDoC	n = 19
Age (y), mean	45 (18–73)
Male n (%)	15 (79)
Diagnosis (0–8 mfi)	10 VS/UWS 9 MCS
Diagnosis at follow up (9–18 mfi)	3 VS/UWS 11 EMCS
Etiology (additional evaluation)	
Traumatic	4 (4)
Anoxic	8 (5)
Vascular	7 (5)
HC	n = 12
Age (y) mean	40 (26–55)
Male n (%)	4 (33)

Report of the demographic and etiology characteristics of the cohort analyzed (*mfi* months from injury).

### Plasma BDNF as biomarkers of pDoC

**Figure 1:**
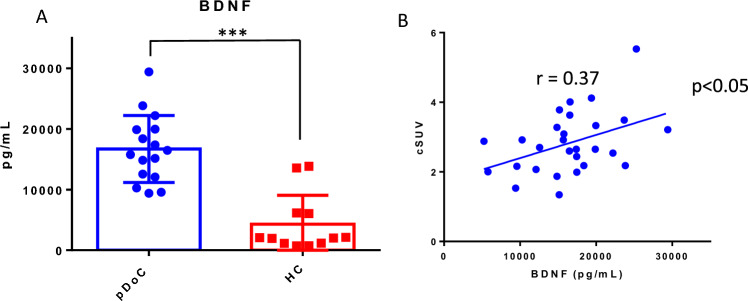
BDNF (**A**) circulating biomarkers in pDoC patients and correlation analysis with cSUV (**B**). The values of the selected marker were obtained from two independent measurements. *p value < 0.05. **pvalue < 0.01. ***p value < 0. 001. *pDOC* prolonged disorders of consciousness, *HCs* healthy controls, *cSUV* cranial SUV, *BDNF* brain derived neurotrophic factor.

To estimate a possible application of BDNF protein as peripheral blood marker of the diagnosis (0–8 months from injury) and/or prognosis (9–18 months from injury) of pDoC, we have determined its concentration in the plasma samples obtained from patients. The plasma concentration of BDNF was significantly higher in pDoC compared with HC (p < 00.5; mean pDoC = 16,696 pg/ml; mean HC = 4254 pg/ml) (Fig. 1, panel A). Also, we observed a positive correlation between BDNF and cSUV (p < 0.05; r = 0.37), suggesting a higher peripheral blood BDNF value in pDoC patients with a higher cranial glucose metabolic activity (Fig. 1, panel B).

### CAMs soluble proteins as potential biomarkers of endothelial dysfunction

**Figure 2:**
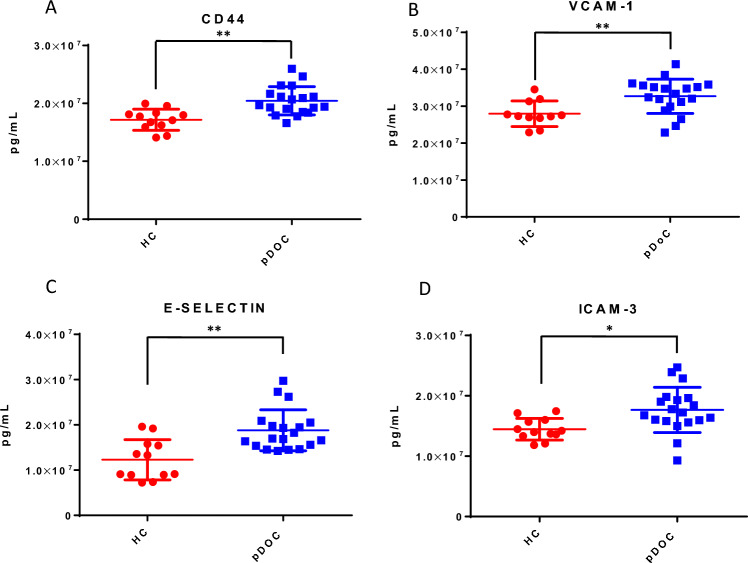
CD44 (**A**), VCAM-1 (**B**), E-SELECTIN (**C**), ICAM-3 (**D**) circulating biomarkers in pDoC patients compared with healthy controls. The values of the selected marker were obtained from two independent measurements. *p value < 0.05. **p value < 0.01. ***p value < 0. 001. *HCs* healthy controls, *pDOC* prolonged disorders of consciousness.

**Figure 3:**
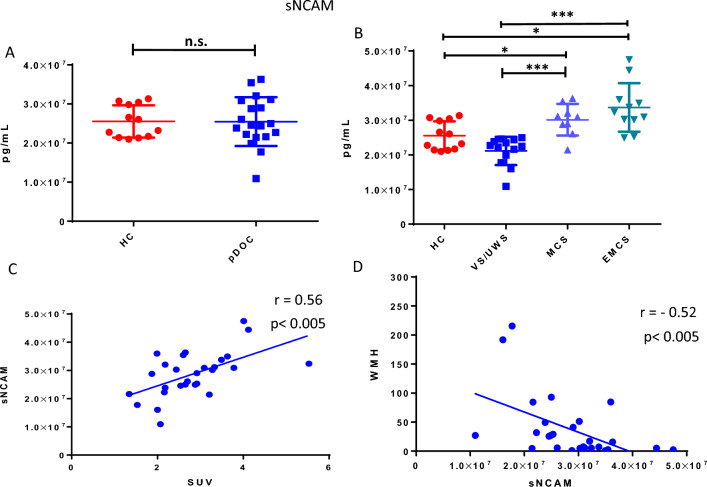
sNCAM circulating biomarkers in pDoC patients compared with healthy controls (**A**) and sNCAM circulating biomarkers in pDoC patients’ subgroups (**B**). Correlation analyses of sNCAM with cranial SUV (**C**) and WMH (**D**). The values of selected marker were obtained from two independent measurements p value < 0.05; **p value < 0.01; ***p value < 0. 001. *SNCAM* soluble NCAM, *HCs* healthy control, *pDOC* prolonged disorders of consciousness, *VS/UWS* vegetative state/unresponsive waking syndrome, *MCS* minimally conscious state, *EMCS* emerged from a minimally conscious state.

We evaluated 13 soluble CAM proteins to study a possible peripheral endothelial dysfunction. Regarding CAMs proteins analyzed, 4 of 13 such as CD44, Vcam-1, E-Selectin, and Icam-3 showed a higher value in pDoC patients compared to HC (Fig. 2, panel A–D); instead, no statistically relevant values were observed for the other protein CAMs analyzed, also stratifying the analyzed cohort (Supplementary Fig. 2). Soluble Ncam, the unique investigated neuron-derived protein, did not show statistically significant differences comparing the pDoC and HCs subgroup (Fig. 3, panel A). Nevertheless, we observed a potential use in the clinical evaluation, stratifying pDoCs patients by clinical diagnosis. Indeed, we observed a statistically significant difference between the VS/UWS subgroup compared to the MCS subgroup (p < 0.0005) and the EMCS subgroup (p < 0.005); no significant difference was observed for the VS/UWS subgroup compared to the HCs as well as for the MCS and EMCS subgroups (p < 0.005) (Fig. 3, panel B).

### Ncam soluble protein: correlation with WMH, SUV and CRSs

To better investigate the role of soluble Ncam, we studied its release into the bloodstream by correlating it with imaging and clinical data. We observed, differently than the other CAMs analyzed, a positive correlation between cSUV and soluble Ncam (r = 0.56; p < 0.005) (Fig. 3, panel C) and, a negative correlation with the WMH (r = − 0.52; p < 0.005) (Fig. 3, panel D). Furthermore, a direct correlation was observed between blood levels of soluble NCAM and CRSs score (r = 0.647 p < 0.0005) (Supplementary Fig. 3).

### pDoC EEG data

**Figure 4:**
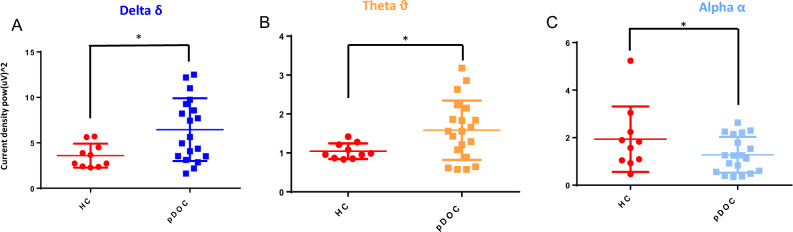
EEG data for delta, theta and alpha waves are shown in blue, orange and sky blue (panel **A**–**C**); significant differences in signal intensity between HCs and pDoC patients are shown (*p < 0.05), with higher values in pDoC patients about delta and theta waves and lower values for alpha compared to HCs. *pDoC* prolonged disorders of consciousness, *HCs* healthy controls.

To further evaluate the pDoC, we analyzed EEG data, comparing pDoC patients with HCs. We observed a statistically significant difference in the analysis of delta, theta, and alpha waves (Fig. 4, panel a–c). Regarding pDoC patients, we observed higher values about delta and theta waves and lower values for alpha compared to HCs. We did not observe significant differences stratifying patients by pDoC stage (Supplementary Fig. 4). Moreover, although a trend towards a positive correlation was observed stratifying patients towards clinical improvement, we observed no correlation between EEG data and soluble NCAM values measured in the blood of pDoC (Supplementary Fig. 5).

## Discussion

BDNF protein displays a pivotal role in the process of brain regeneration upon several types of damages, promoting the resilience of neuronal cells against neurodegeneration^21^. Our pilot study identified BDNF protein as a possible diagnostic marker by comparing patient pDoCs with healthy controls (mean pDoC = 16,696 pg/ml; mean HC = 4254 pg/ml). It has also been observed that BDNF protein correlates with cSUV, making it a candidate as a possible alternative marker when PET imaging is not possible. Disorders of consciousness are known to be associated with blood–brain barrier (BBB) damage. Although the mechanism underlying damage to the BBB is not clearly understood, a disruption of the BBB is associated with the activation of inflammatory pathway and the activation of endothelial cells that can express increasing numbers of surface antigens. In our study, we hypothesized that blood detection could provide insights into improved clinical management of patients with pDoC. Among the 13 CAMs analyzed, 4 out of 13, such as the soluble CAM proteins CD44, Vcam-1, E-Selectin, and Icam-3, were found to be significantly higher in pDoCs compared to HCs (p value < 0.01 for CD44, Vcam-1, E-Selectin, and p value < 0.05 for Icam-3 respectively)^28–31^. Excluding EPCAM and L-selectin, the other CAMs analysed also show an increasing trend in pDOC patients compared to controls, which could become significant by increasing the study cohort. This confirms that in pDOC patients could be in progress processes of neuroinflammation, extracellular matrix reorganization, and immune system cell activation which are a direct consequence of brain injury. Furthermore, a potential use for the soluble NCAM protein was identified, as it can discriminate between the VS/UWS subgroup compared to the MCS subgroup (p < 0.001) and correlates with the cSUV (r = 0.56; p < 0.005) and WMH (r = − 0.52; p < 0.005). Bagnato et al.^9^ observed that serum BDNF levels in n = 18 pDoC patients and n = 16 HCs (range 1–7.3 months after brain injury) were reduced in pDoC patients compared to HCs, also demonstrating that the level of BDNF was not modified by verticalization with robot-assisted stepping. In our study, we analyzed plasma samples obtained from n = 19 pDoC patients and n = 12 HCs, evaluating a broader interval of 1–18 months after brain injury, observing a higher value of BDNF in pDoC patients (mean = 16,696 pg/ml) compared to HCs (mean = 4254 pg/ml). Moreover, our technical approach was different because Bagnato et al. used the PicoKine™ enzyme-linked immunosorbent assay kit, while in this study we used the Simoa technology (Quanterix, Billerica, USA). However, our approach included a neuroimaging investigation, evaluating the presence of brain lesions by magnetic resonance imaging (MRI), the evaluation of cerebral metabolism of glucose by PET imaging and analyzing EEG data together with soluble CAM proteins. Although EEG data can separate pDoC from HCs, they cannot discriminate stratifying patients by pDoC stage. Furthermore, the absence of correlation with blood parameters does not allow the EEG to be used as a parameter to be integrated with circulating biomarker levels in pDoC. On the other hand, the relationship between imaging parameters and soluble CAM proteins has not been explored in pDoC patients, to the best of our knowledge. Our attention was focused on the soluble form of Ncam, particularly useful in stratifying pDoC patients. Indeed, it positively correlated with cSUV and negatively with the WMH parameter, associated with lesion size, suggesting a possible application together or as an alternative when imaging it is not possible to evaluate the brain lesion recovery. As known, soluble NCAM can modulate neurite outgrowth and branch in vitro, processes presumably governed by complex dose–response relationships^28^. It knows that its overexpression in mice appears to perturb synaptic connectivity and outcomes in abnormal behavior. The release of soluble NCAM may be a biological mechanism that controls cell adhesion and regulates aspects of synapse formation and function, but this aspect has not been fully elucidated^12,32^. Although our cohort has been well characterized about clinical, imaging, and blood marker data, it is essential to underline that the sample size does not allow us to generalize our results. However, while our data are interesting, there is a need to validate the observations on a larger study cohort to generalize the observations and make the obtained data useful for improving the management of the pDoC patient. Furthermore, despite we had verified that the molecules analyzed were not sex-dependent (data not shown), there is an imbalance with a greater presence of male subjects in the cohort, a known bias in neuroscience research^33^.

The pDoC patients’ clinical management is very difficult as they are often connected to medical instruments that preserve vital signs and therefore it is not easy to obtain complete medical data. To this end, as indicated in our manuscript, the possibility of dosing innovative circulating biomarkers that can provide clinical information not only of neuronal damage but also of recovery of cognitive functions, becomes crucial to generate new diagnostic approaches that are more effective in the management of this acute neurological pathology.

## Conclusion

Albeit preliminary, our data introduce BDNF protein as a possible marker of patients with pDoC and the soluble CAM markers CD44, Vcam-1, E-selectin, and Icam-3 as markers of diagnosis of pDoC. Furthermore, the possible use of the soluble Ncam protein in the evaluation of pDoC patient’s prognosis hypothesizes a possible role of this protein in the recovery and rearrangement of neuronal plasticity to be investigated. Finally, it was highlighted that correlation with imaging data could be a key approach to the clinical management of DoC. The support of professionals such as radiologists, biomedical engineer, biologists to the clinical team is increasingly crucial to obtain more information and to better characterize this specific population for diagnosis and prognosis.

### Supplementary Information

Supplementary Information.

## Data Availability

The datasets used and analyzed during the study are available from the corresponding author on a reasonable request.

## Abbreviations

^18^F-FDG: ^18^F-fluorodeoxyglucose
BDNF: Brain-derived neurotrophic factor
CAMs: Cell adhesion molecules proteins
CAMs: Soluble cell adhesion molecules
CRS-R: Coma recovery scale-revised
cSUV: Cranial standardized uptake value
EMCS: Emerged from a minimally conscious state
EEG: Electroencephalography
FA: Fractional anisotropy
GFAP: Glial fibrillary acidic protein
HCs: Healthy controls
LPA: Lesion prediction algorithm
L-selectin: Leukocyte selectin
LV: Lesion volumes
MCS: Minimally conscious state
mfi: Month from injury
NF-L: Neurofilament light chain
OSEM: Ordered subset expectation maximization
pDoC: Prolonged disorders of consciousness
P-selectin: Platelet selectin
Psgl-1: P-selectin glycoprotein ligand-1
sIcam-1: Soluble intercellular adhesion molecule-1
sVcam-1: Soluble vascular cell adhesion molecule-1
TBI: Traumatic brain injury
VS/UWS: Vegetative state/unresponsive waking syndrome
WMH: White matter hyperintensities
