# Supplementary material for: Peripheral blood BDNF and soluble CAM proteins as possible markers of prolonged disorders of consciousness: a pilot study
Source: Sci Rep. 2024 Jan 3;14:341. doi: 10.1038/s41598-023-50581-8 (PMC10764320; doi:10.1038/s41598-023-50581-8)
Supplement: Supplementary file 1 — Supplementary Information. [file 41598_2023_50581_MOESM1_ESM.pdf]

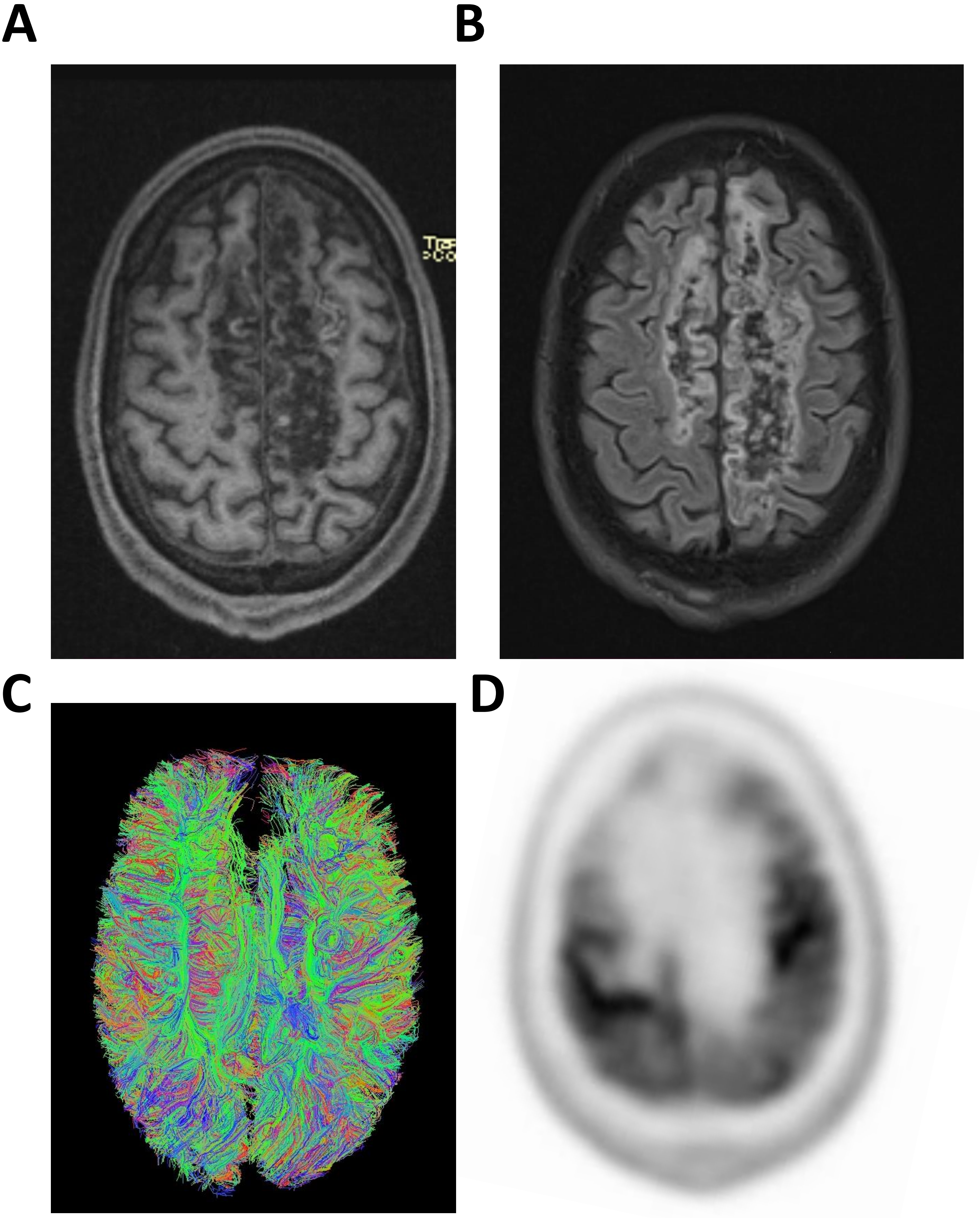

Supplementary Figure 1. A 37 y-o MCS+ male patient with a bilateral vascular lesion of medial cortices of both frontal lobes. In (A), axial T1-weighted MR showing a bilateral cortical frontal lesion, more extended in the left hemisphere. In B, an axial FLAIR image showing a gliotic boundary for both lesions. In (C), a superior view of the whole-brain tractography reconstruction showing a lack of streamlines in the medial part of frontal lobes, and a direct view of both the cingulum tracts. In (D), an axial FDG-PET image showing a diffuse hypometabolism of the injured cortices. In (E) we show in red circle the BDNF value of the pDoC patient represented with imaging.

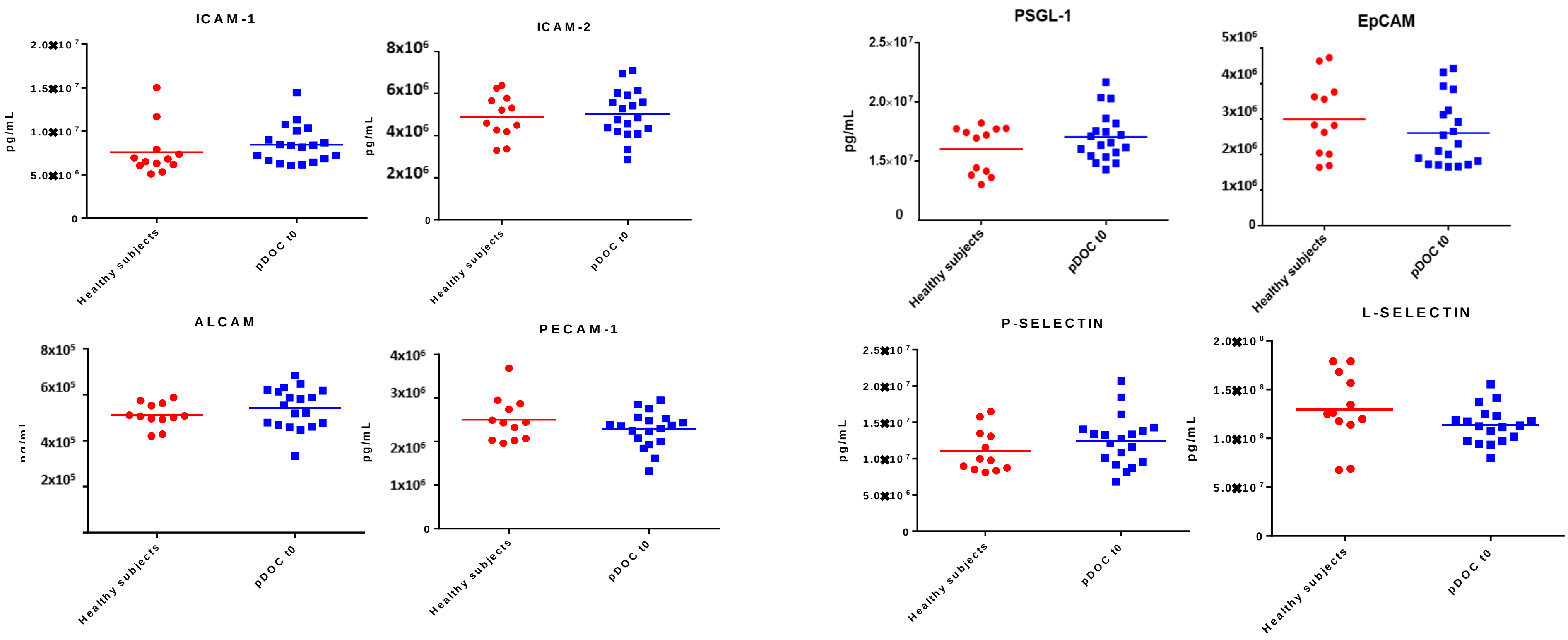

Supplementary Figure 2: Soluble ICAM-1, ICAM-2, ALCAM, PECAM-1 (A) and PSGL-1, EpCAM, P-Selectin, L-Selectin (B) circulating biomarkers in pDoC patients compared with healthy controls. No significant differences were observed between two subgroups.

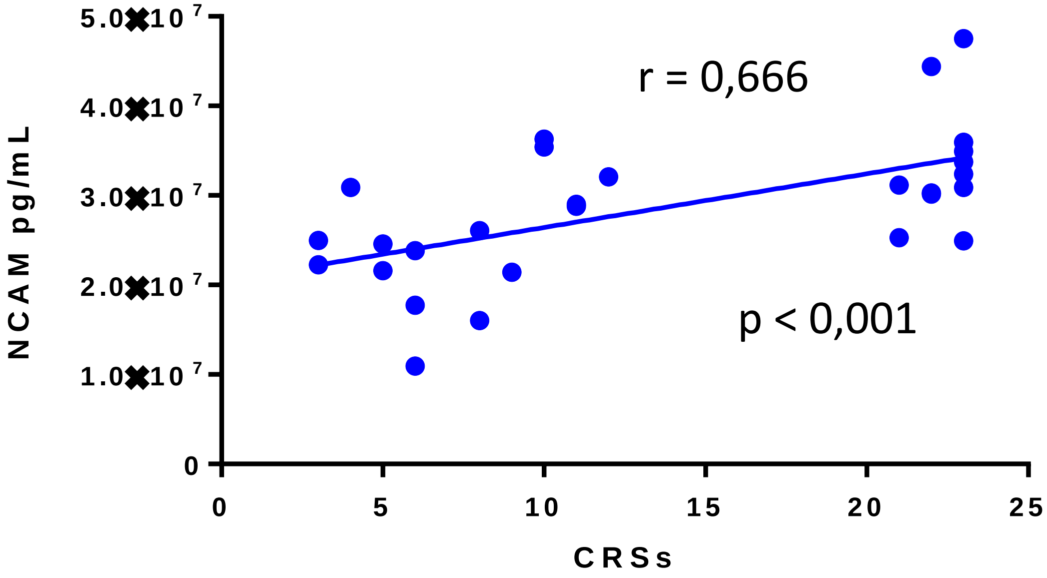

Supplementary Figure 3: Correlation between soluble NCAM protein and CRSs (r = 0,66; *** = p-value< 0. 001).

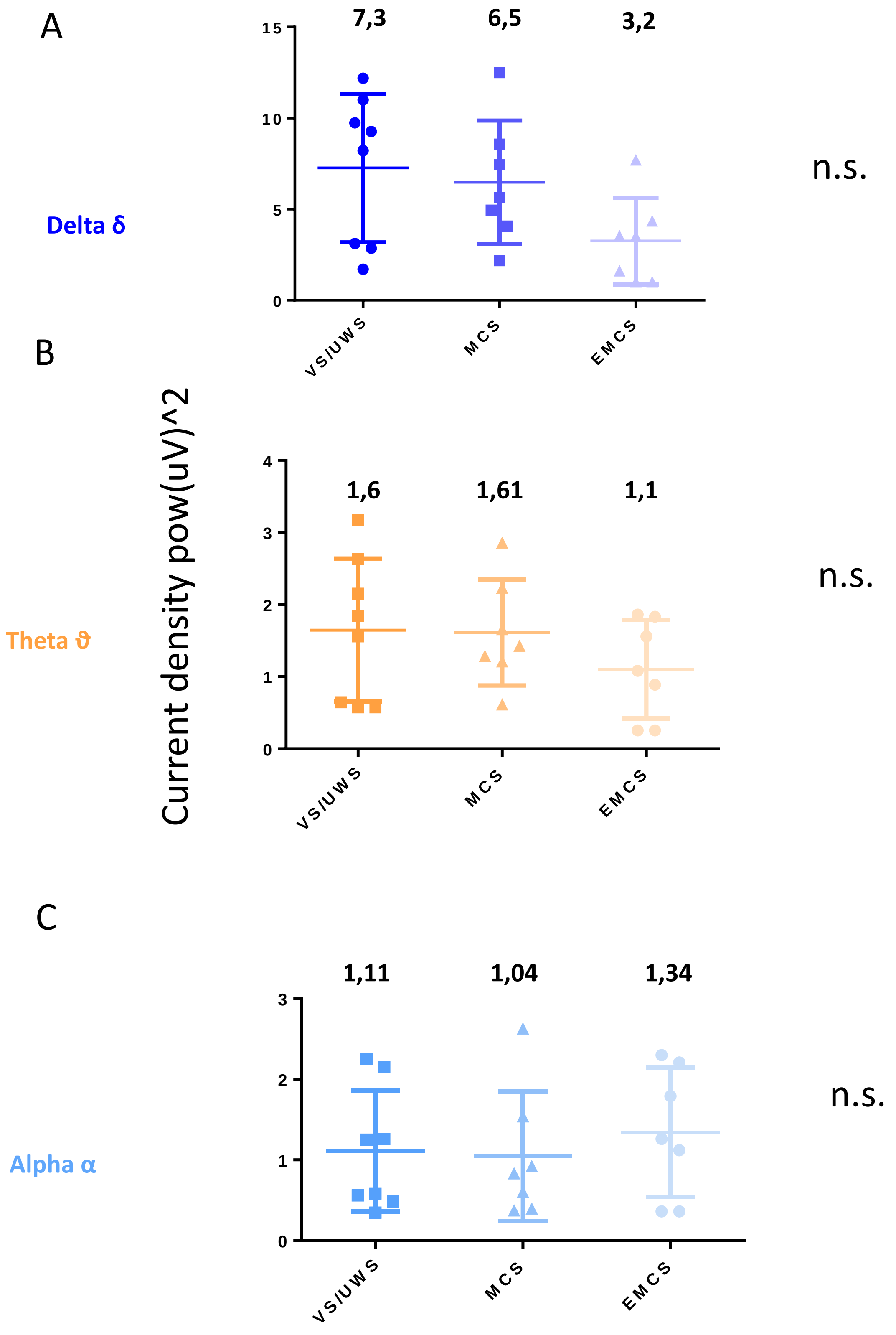

Supplementary figure 4: EEG data analysis. The EEG data for delta waves are shown in blue (panel A); theta waves are shown in orange (panel B) while alpha waves are shown in blue (panel C). Mean data related to delta, theta e alpha are showed regarding VS/UWS, MCS and EMCS subgroups. VS/UWS = Vegetative State/Unresponsive Waking Syndrome; MCS = minimally conscious state; EMCS = emerged from a minimally conscious state.

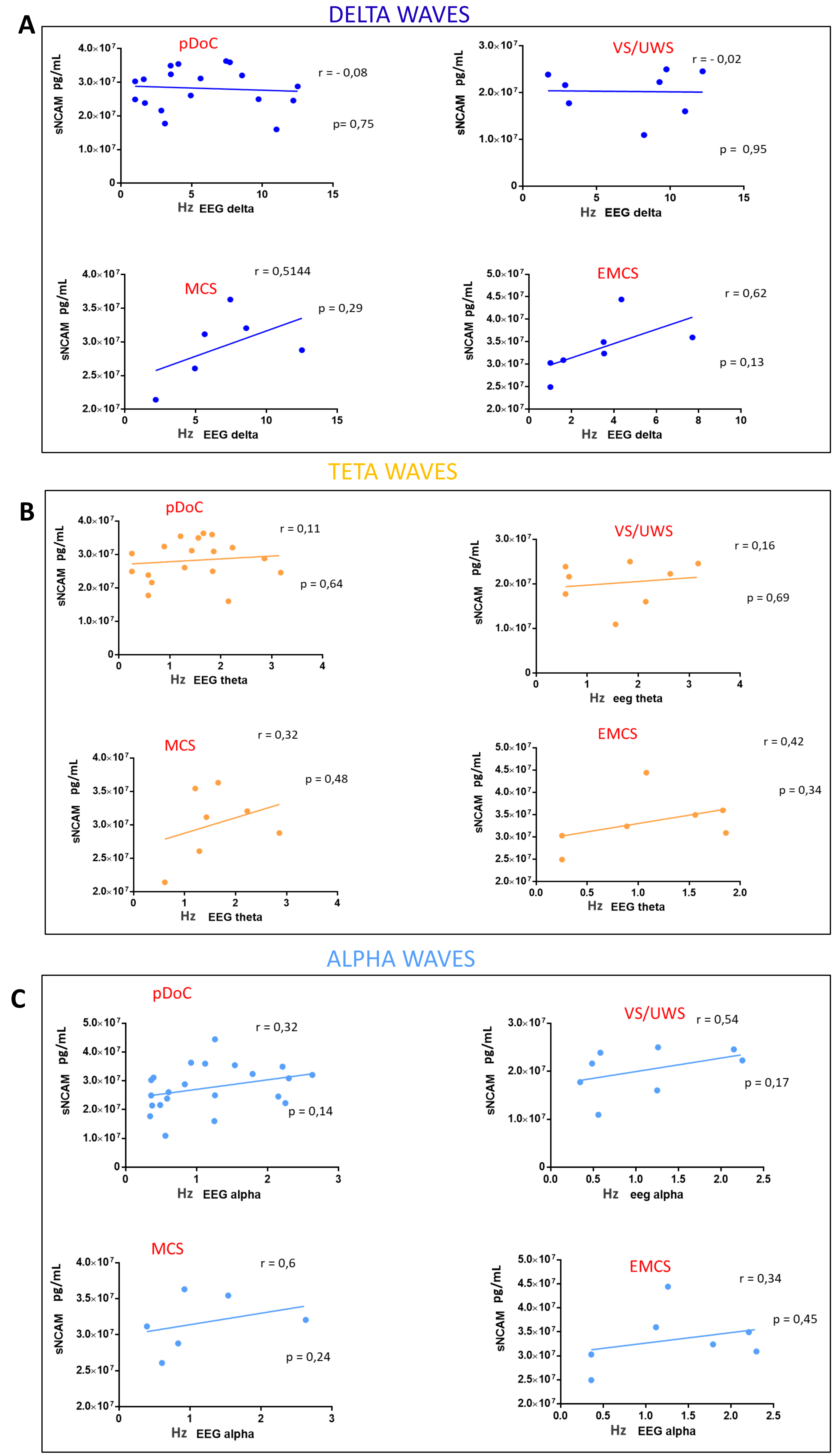

Supplementary figure 5: Correlations of EEG data with sNCAM level. Delta waves (A), Teta waves (B) and Alpha waves (C) in pDoC patients and in VS/UWS, MCS and EMCS subgroups. pDoC = prolonged disorders of consciousness; MCS = minimally conscious state; EMCS = emerged from minimally coscious state
